# Supplementary material for: Are carbon emissions trading and green financial instruments synergistic? -Comprehensive quantitative research based on content analysis
Source: PLoS One. 2024 Mar 7;19(3):e0298601. doi: 10.1371/journal.pone.0298601 (PMC10919601; doi:10.1371/journal.pone.0298601)
Supplement: S1 Data — (ZIP) [file pone.0298601.s001.zip › Data/地级市Z_F工作报告.zip!/╣π╬≈/║╪╓▌╩╨/└┤╫╘╣1⁄2╓┌║┼í╛┬φ┐╦╩2╛▌═°í┐_2018.docx]

各位代表:

　　现在,我代表贺州市第四届人民政府向大会作工作报告,请予审议,并请市政协委员和其他列席人员提出意见。

　　一、2017年工作回顾

　　2017年,是贺州发展极不平凡的一年,面对经济发展新常态下的严峻困难和挑战,在自治区党委、政府和市委的正确领导下,在市人大、政协的监督支持下,我们深入学习贯彻落实党的十九大精神,以习近平新时代中国特色社会主义思想为指导,紧紧围绕市四届人大二次会议确定的目标任务,迎难而上,开拓进取,奋发有为,推动经济社会发展稳中提质、稳中增效。预计全年地区生产总值540亿元,同比增长5.5%；财政收入53.1亿元,增长4.3%；固定资产投资720亿元,增长10.6%；社会消费品零售总额178.3亿元,增长10.8%。

　　——新旧动能加快转换。坚决贯彻国家“去产能”政策,处置生产“地条钢”企业8家,彻底拆除中频炉等生产“地条钢”设备及附属设施,累计拆除中频炉52套104台,涉及产能340.9万吨。碳酸钙产业发展壮大,全年产值突破200亿元,同比增长25%,成为全国最大的人造岗石生产基地,获“中国贺州·岗石之都”称号。装配式建筑产业迈出新步伐,有9个生产基地落地建设,其中6个申报自治区级试点项目。

　　——“长寿·养生”经济健康发展。设立广西姑婆山森林生态养生旅游产业区,荣获“全国森林旅游示范市”“中国十大养生城市”称号,昭平南山茶海景区、黄姚花海景区获评为国家4A级旅游景区,全市接待旅游总人数、旅游总消费分别增长18.7%和25.1%。贺州被列为全区唯一一个建设“中医壮瑶医药养生保健示范基地”的地级市。现代特色农业示范区建设成效显著,实现自治区级创建县(区)全覆盖,建成广西最大的黑木耳生产基地。

　　——开放合作持续深化。设立贺州临港产业区,永贺高速主线建成通车,贺巴高速贺州段顺利推进,开放大通道枢纽逐步成型。启动贵广高铁经济带合作示范区建设,与粤桂黔高铁沿线的12个市(州)共同签署《粤桂黔高铁经济带协同创新合作共识》。加快融入粤港澳大湾区,引进贺州通用航空产业园和姑婆山体育文化旅游等重大项目216个,总投资额达221.7亿元；成功举办首届粤港澳大湾区高层次人才寿城行活动,发布粤港澳大湾区高层次人才贺州倡议。

　　——城乡面貌明显改善。宜居城市建设扎实推进,市文化中心投入使用,贺州园博园、爱莲湖公园实现开园,北堤公园二期、贺州广场完成提升改造,城东新区路网、桥梁建设和灵峰山以西旧住宅综合整治工程一期基本完成。成功举办第十届广西(贺州)园林园艺博览会。乡村人居环境持续改善,“生态乡村”建设通过自治区验收；“宜居乡村”建设成效明显,农村新型生态社区建设经验在全区全国推广,钟山、富川被确认为首批全国农村社区治理实验区；昭平再次获评为“中国百佳深呼吸城市”,黄姚荣获“第二批全国特色小镇”称号,富川6个村(社区)入选全国第一批绿色村庄。

　　——民生水平稳步提升。预计城镇居民人均可支配收入和农村居民人均可支配收入分别增长8%、10%,达到29034元和10507元。累计开工建设保障性住房、棚户区和农村危房改造近1.1万套(户)。市中医医院附属护理医院、第五人民医院已投入使用。绿色交通发展迈上新台阶,投放新能源纯电动公交车200辆,成为广西首个城区实现纯电动公交车全覆盖的地级市。

　　一年来,我们主要做好以下工作:

　　一是坚持目标引领,脱贫攻坚扎实推进。筹措落实财政扶贫资金17.4亿元,全力打赢脱贫攻坚战。经市级初审,实现了八步区脱贫摘帽,49个贫困村出列,6.01万贫困人口脱贫。易地扶贫搬迁项目全部实现开工,完成住房9533套,搬迁入住41181人。创新贫困村集体经济增长方式,形成资产盘活模式、茶园模式和光伏模式,脱贫的49个贫困村集体经济收入全部达到2万元以上。贫困村基础设施建设短板加快补齐,解决有安全饮用水2159户、有电用1010户,完成村屯通路596.1公里。教育扶智、医疗救助、低保兜底政策全面实施。

　　二是坚持迎难而上,经济基本面保持平稳。将稳增长摆在更加突出的位置,开展稳增长“六大行动”,建立主要经济指标“红黑榜”,推动经济持续平稳发展。克难攻坚稳工业,碳酸钙产业支撑能力进一步增强,占全市规模以上工业总产值比重达35%；装配式建筑产业加快推进,超超新材装配式建筑产品已投放市场,贺州通号、广西建工等装配式建筑项目开工建设；电子新材料产业链不断延伸,正润日轻高纯铝、正润新材料项目实现投产。全年降低企业成本7.2亿元。全力以赴稳农业,粮食总产量达70.7万吨；通过区、县、乡三级现代特色农业示范区验收14个；钟山县入选第一批国家农业可持续发展试验示范区,昭平县荣获“全国十大魅力茶乡”称号。大力发展服务业,扎实推进“十三五”国家服务业综合改革试点市建设,深入实施“服务业综合改革专项行动”,年内入库服务业企业39家,预计服务业增加值占GDP比重达40.7%,对全市经济增长贡献率超过70%。全市存款余额722亿元,增长16.4%,贷款余额455亿元,增长26%。发展壮大生态健康旅游产业,大力推进国家级医养结合试点市建设,姑婆山、东潭岭、园博园等医养结合项目扎实推进,黄姚获评首批“广西养生养老小镇”；积极开展国家全域旅游示范区创建工作,姑婆山旅游区创建国家5A级景区、昭平创建广西特色旅游名县工作有序推进；成功举办第三届中国(贺州)新媒体群英会暨广西贺州第二届长寿文化节、第二届中国—东盟商会领袖高峰论坛贺州分论坛、贺州长寿高峰论坛、第三届两岸青年农业论坛。充分激发非公经济活力,落实厅级领导联系服务非公企业制度,组建非公经济发展服务中心,强化政企对接会的作用,推动非公经济“两个健康”发展。

　　三是坚持问题导向,重大项目建设成效明显。成立“10+2”重大事项、重大项目工作领导小组,对重点产业、项目实行清单管理,压实“一把手”抓项目抓投资责任。千方百计上项目、扩投资,382项市级层面统筹推进重大项目完成投资259亿元,49项自治区层面统筹推进重大项目完成投资63.9亿元,均超额完成年度任务；中央预算内投资项目投资完成率荣登全区稳增长红榜；启动贺连高速、贺州临港产业区一期基础设施等PPP项目,钟山碳酸钙改性母粒、广厦环保科技等一批重大项目开工建设,纯一陶瓷等一批重大项目建设加快,华砻树脂、力丰实业等一批重大项目实现竣工投产。着力保障项目用地,落实用地指标1.3万亩,对扶贫项目、自治区统筹推进重大项目做到应保尽保。全面提升项目审批效率,项目联合审批信息管理平台共受理办结审批事项1836件,办结提速53.3%。着力破解项目融资难题,工业发展、旅游发展等5支基金到位52亿元；争取自治区支持新增政府债券22.1亿元,全市银行业金融机构各项贷款新增90亿元,增长24.2%,融资总量实现新突破。

　　四是坚持突出重点,改革创新深入实施。启动九大领域45项重点改革任务。“多规合一”试点取得新进展,《贺州市空间规划(2016—2030年)》成为全国第一个获批的空间规划。园区体制机制改革不断深化,出台工业园区竞聘上岗、薪酬收入分配和绩效考评三个配套文件,吸纳更多优秀人才服务园区发展。深化国资国企改革,发展壮大市级6个平台公司,统筹编制平台公司发展规划。农业农村改革迈出新步伐,农村土地确权登记颁证工作可发证率达97.4%,全区排名前列。深化“放管服”改革,扎实推进权责清单“两单融合”工作,核定、保留事项321项,取消事项47项。大力推进商事制度改革,推行“多证合一、一照一码”登记制度,以“减证”推动“简政”。在全区首创推行“农金村办”助农惠农补贴一卡通系统。优化公共资源交易流程,推进交易电子化,完成交易项目2488项,节约资金3.3亿元。稳妥推进事业单位分类改革,乡镇“四所合一”改革通过自治区评估验收。科技创新能力进一步增强,贺州电子新材料等4家产业研究院正式挂牌,微波应用技术重点实验室组建揭牌,组建广西人造石材工程技术研究中心1家,新增高新技术企业5家,获批国家级“众创空间”1家、“星创天地”2家。

　　五是坚持对外开放,开放发展水平稳步提升。深入实施向东开放战略,积极构筑开放新高地。加快完善园区基础设施,重点产业园区基础设施完成投资29亿元,增长20.8%。出台《贺州市关于加快工业园区经济发展的若干政策措施(试行)》,促进园区产业集聚集群发展。大力实施精准有效招商,加强与高铁经济带合作实验区广东园、贵州园的交流合作,引进贺州(深圳)智慧产业园·长寿谷等重大项目；成功举办第二届中国(贺州)石材·碳酸钙展览会,签约项目24个,达成投资金额47亿元；成功在港澳举办广西贺州投资推介会,签约投资5.3亿美元。全市招商引资实际到位资金410亿元,同比增长14.6%。

　　六是坚持绿色发展,生态宜居环境持续优化。城区基础设施进一步完善,城东新区、“一江两岸”、高铁新城等重大项目加快建设。以建市十五周年为契机,扎实推进64个市庆项目,累计完成投资39.8亿元；贺州大道、光明大道等城区主干道提质改造基本完成,贺州大桥、灵峰大桥等夜景全部亮化,完成建设路、平安西路等15条道路的绿化景观提升改造,市容市貌明显改观。加强生态保护和治理,贺州生态保护与建设示范区入选国家典型示范区,钟山县荣获“2017绿色中国特别贡献奖”,贺州市循环经济环保产业园等设施建设加速推进；饮用水源水质、主要江河水库功能区水质达标率达100%,率先在全区推行“河长制”河道管理模式,构建市、县、乡、村四级联动河长体系。全力抓好中央环境保护督察反馈意见的整改。

　　七是坚持以人为本,社会民生事业加快改善。全力保障和改善民生,民生领域支出150.9亿元,占一般公共预算支出的83.1%。教育事业加快发展,投资17亿元实施988个教育基础设施项目建设,开工建设黄姚职业教育中心,启动贺州学院黄姚校区、市中心城区8所新(迁)建中小学(幼儿园)项目建设,钟山县、富川瑶族自治县通过国家级县域义务教育均衡发展评估验收。地方文化繁荣发展,黄姚产业区文化中心开工建设,潇贺古道在“2017中国十大古道”评选中成功入选为最具地方特色古道,电视剧《外来媳妇本地郎》、纪录片《潇贺古道》、电影《别在村前等我》播出反响强烈,《瑶山秀彩云》《江山交付的下午》等一批文艺文化作品获国家相关部门奖励。扎实推进基层就业和社会保障服务建设,城镇新增就业1.26万人,农村劳动力转移新增就业2.78万人次；城乡居民养老保险和基本医疗保险参保率均超额完成年度任务。加强全面治理拖欠农民工工资问题工作。不断深化医药卫生体制改革,所有公立医院取消了15%的药品加成,医联体分级诊疗试点工作实现县(区)全覆盖,顺利通过广西卫生城市复检,在全区率先完成国家流动人口卫生计生动态监测工作；市人民医院城东分院、市妇幼保健院搬迁、黄姚产业区医院等项目加快推进。大力开展全民健身活动,成功举办中国龙舟公开赛(广西·昭平)、首届桂湘粤港澳台足球邀请赛(广西·钟山)等赛事,“寿城·贺州”万人吸氧操成功挑战吉尼斯世界纪录,贺州市体育局获“全国体育系统先进集体”称号。广西食品安全城市创建工作深入推进。民族团结进步创建活动深入开展,在全区率先启动民间信仰管理试点工作,富川瑶族自治县的深坡、福溪、虎马岭三个传统村落被命名为“中国少数民族特色村寨”。切实抓好诚信贺州、法治贺州、平安贺州建设,钟山县公安局荣获“全国优秀公安局”称号,圆满完成党的十九大安保维稳工作。

　　同时,安全生产、信访、防灾、气象、人防建设等方面得到加强,妇女儿童、老年人和残疾人权益得到有效保障,社会救助制度日趋完善,侨务、群团组织、“扫黄打非”等工作和供销、农机、二轻、档案、史志等事业取得新成绩,双拥共建、军民融合工作迈出新步伐。

　　一年来,我们坚持以全面从严治党统领政府各项工作,依法全面履行政府职能,努力打造人民满意政府。加快推进法治政府建设,坚持依法行政,落实司法建议,加强和改进行政复议和行政应诉工作,完善政府法律顾问制度,加强法治政府队伍建设。提升政府服务效率,推行政务服务集中办理,按时办结率达100%。坚决服从市委的领导,自觉接受市人大、政协监督,人大代表建议和政协委员提案办结率均达100%。推动“两学一做”学习教育常态化制度化,大力开展忠诚观教育,深入学习贯彻落实党的十九大精神,全面贯彻落实中央八项规定精神和国务院“约法三章”,不断加强重大项目、重点行业企业的审计监督和行政监察,“三公”经费缩减12.7%。

　　各位代表,一年来,我们顶住了压力,经受住了考验,取得的成绩来之不易。这是自治区党委、政府正确领导和深切关怀的结果,是市委统揽全局科学决策的结果,是市人大、政协和社会各界监督支持的结果,是全市各级干部和群众团结拼搏、开拓奋进的结果。在此,我谨代表市人民政府,向全市人民,向人大代表、政协委员、各民主党派、工商联、无党派人士和各人民团体,向驻贺部队、武警官兵,向所有关心支持贺州发展的海内外朋友,表示衷心的感谢并致以崇高的敬意!　(下转第二版)

　　(上接第一版)在充分肯定成绩的同时,我们也清醒地看到前进中还面临不少问题和困难。主要是:稳增长压力大,特别是受国家“去产能”政策影响,工业转型升级困难较大,产业基础薄弱单一,结构不合理,传统资源型产业比重大；区域发展不平衡不充分,县域经济发展滞后；工业园区和市管国有企业发展活力不强；项目融资渠道收窄；基础设施和公共服务供给不足,脱贫攻坚任务艰巨,民生短板突出；开放质量水平整体不高；政府职能转变步伐仍不够快,机关作风还需改进,营商环境有待改善。困难孕育着生机,改革发展已经破题。为此,我们要直面困难,迎难而上,以改革创新的精神不断破解难题,打开工作新局面。

　　二、2018年工作安排

　　今年是全面贯彻党的十九大精神的开局之年,是改革开放40周年,是决胜全面建成小康社会、实施“十三五”规划承上启下的关键一年,又是自治区成立60周年。当前,贺州发展正处于可以大有作为的重要战略机遇期。粤港澳大湾区建设上升为国家战略,有利于我市对外开放合作、承接产业转移,加快实现发展新定位；国家提出实施乡村振兴战略、自治区提出实施县域经济三年行动计划等机遇,将为加快我市城乡一体化进程提供有力支撑；我市“十三五”国家服务业综合改革试点工作作为推进全区服务业发展的重要载体,政策红利将进一步释放,助推全市服务业发展壮大；我市荣获广西首个“全国森林旅游示范市”称号,将更有效地把生态资源优势转化为生态健康产业发展的强大动力。我们必须树立强烈的机遇意识和赶超意识,坚定信心,振奋精神,鼓足干劲,不断开创贺州改革发展新局面。

　　2018年政府工作的总体要求是:全面贯彻党的十九大和中央经济工作会议精神,以习近平新时代中国特色社会主义思想为指导,坚持稳中求进工作总基调,坚持新发展理念,紧扣社会主要矛盾变化,按照高质量发展的要求,统筹推进“五位一体”总体布局和协调推进“四个全面”战略布局,坚持以供给侧结构性改革为主线,统筹推进稳增长、促改革、调结构、惠民生、防风险各项工作,充分发挥“世界长寿市”的品牌优势,以推动大创新、实施大开放、培育大物流、发展大旅游、构建大健康“五大新引擎”为抓手,推动质量变革、效率变革、动力变革,深入实施“五大战略”和“五大攻坚行动”,促进经济社会持续健康发展,为加快实现发展新定位、全面建成小康社会、谱写新时代贺州发展新篇章奠定坚实基础。

　　根据上述总体要求,经济社会发展主要预期目标是:地区生产总值增长8%,财政收入增长6%,规模以上工业增加值增长8%,固定资产投资增长12%,社会消费品零售总额增长11%,进出口总额增长10%；居民消费价格涨幅控制在3%以内,城镇居民人均可支配收入和农村居民人均可支配收入分别增长9%和10%,城镇登记失业率控制在4.2%以内,常住人口城镇化率达48%,节能减排降碳指标完成自治区下达任务。

　　为更好地实现上述目标,市委四届六次全会明确提出了推动大创新、实施大开放、培育大物流、发展大旅游、构建大健康“五大新引擎”。“推动大创新”,主要是以开展“多规合一”试点市、国家级医养结合试点市、国家全域旅游示范区等改革试点工作为契机,加快在产业发展思路、体制机制、关键技术上创新。“实施大开放”,主要是充分发挥我市作为广西“东大门”、毗邻粤港澳大湾区的区位优势,进一步按照自治区赋予贺州的发展新定位,打造高水平开放平台、打通开放通道、抓好招商引资。“培育大物流”,主要是针对我市目前物流成本太高的现状,以发展现代综合交通运输体系为支撑,大力发展现代物流业。“发展大旅游”,主要是充分发挥我市优质的旅游和生态资源以及“世界长寿市”“中国十大养生城市”的品牌优势,以高水平创建国家全域旅游示范市为契机,以黄姚古镇旅游文化产业区、姑婆山森林生态养生旅游产业区等建设为重点,构建观光与生态、休闲、体育、养生结合,旅游与文化、农业、工业融合的大旅游产业链。“构建大健康”,主要是依托粤港澳大湾区巨大的消费市场,推动大健康与大生态、大旅游深度融合,发展“长寿·养生”经济,推进健康贺州建设,打响“寿城”品牌。“五大新引擎”的提出,是推动我市经济社会持续发展的重要遵循和有力抓手。为全面贯彻落实好市委“五大新引擎”的科学部署,我们将重点抓好以下七个方面的工作:

　　(一)以更大决心打好精准脱贫攻坚战。坚持精准扶贫、精准脱贫,强化责任落实、政策落地、群众动员,巩固脱贫质量,确保实现全年贫困人口脱贫7.15万人、60个贫困村脱贫出列和富川瑶族自治县脱贫摘帽。

　　拓宽产业扶贫渠道。立足农业、旅游、生态、长寿等资源优势,因地制宜选准产业,培育壮大优势特色种养业,大力发展农产品加工,加快发展乡村旅游、休闲农业等新产业新业态,抓好品牌建设。继续推广“三统一分”“以奖代补”等产业扶贫模式,力争每个县(区)发展“5+2”特色优势产业、每个贫困村形成“3+1”主导产业,覆盖60%以上的贫困户。加强扶贫产业的技术、金融、管理和营销等各种配套服务。引进一批龙头企业到贫困村投资发展,尤其要利用“IPO”绿色通道,吸引资本市场助力脱贫攻坚,促进贫困地区产业规模化生产、集约化经营。培育壮大新型经营主体,实现农民专业合作社在267个贫困村全覆盖。继续探索农村集体经济发展的有效途径,加大金融对产业扶贫的支持力度,实现贫困村集体经济收入达到3万元以上。

　　深入推进易地扶贫搬迁。从严落实国家和自治区督查反馈问题的整改工作。严格按照国家易地扶贫搬迁政策新要求,在搬迁补助、拆除旧房奖励、土地支持、搬迁后配套等方面加以倾斜。落实“市包县、县包项目”责任制,加快易地扶贫搬迁项目建设进度,确保完成3289名贫困人口的搬迁任务。有序开展易地扶贫搬迁拆旧复垦试点工作,每个县(区)落实2个以上拆旧复垦示范点。加强搬迁后续扶持工作,统筹做好安置点水、电、路、网等配套基础设施建设,合理布局教育、卫生等公共资源,同步做好产业帮扶、劳务培训和输出等配套工作,确保搬迁群众安居乐业。

　　强化脱贫保障措施。加大各类扶贫资金统筹力度,投入到贫困村基础设施建设。深入贯彻落实广东、广西两省区联合印发的《关于进一步加强粤桂扶贫协作工作的意见》,深化与广东省肇庆市的扶贫协作。大力推进教育扶贫,精准实施学生帮扶计划,完善从学前教育到高中阶段教育的资助体系。大力推进健康扶贫,扩大贫困地区基层医疗机构“先诊疗、后付费”服务模式的实施范围,实施贫困人口新农合个人缴费财政专项补助政策。加强对低保对象和建档立卡贫困人口的动态管理,提高低保标准和覆盖面。深入开展扶贫领域作风问题专项治理,继续实行督查暗访、“红黑榜”通报,加强扶贫资金监管,提高扶贫资金使用效率,严肃查处脱贫工作中出现的贪污挪用、截留和私分问题。

　　(二)以更大力度加快实体经济发展。围绕供给侧结构性改革,开展“实体经济服务年”活动,优化产业结构,促进新旧动能转换,推动高质量发展。

　　推动工业转型升级。全面推进传统优势产业二次创业,改造振兴电力、钢铁加工、有色冶炼等传统特色产业,建设与粤港澳大湾区产业配套的钢铁铸件和不锈钢生产基地,积极打造广西—东盟矿山装备制造基地。做强做优碳酸钙产业,启动“碳酸钙产业转型升级三年行动计划”,推进长城机械技改、利升石业石材家居中心等重点项目建设,加快碳酸钙全产业链发展。运用好自治区装配式建筑试点市政策,发展壮大装配式建筑产业,举办绿色产业发展论坛,探索建立产品质量标准体系和产品应用技术体系；扎实推进贺州“四馆”等装配式建筑试点项目；创建新型装配式产业工人实训基地；加快建设广西建工等装配式建筑生产基地,实现贺州通号装配式建筑生产基地竣工投产。大力发展电子新材料、稀土等高新技术产业,加快推动力达新医药港·贺州生物产业园等项目建设。继续深入开展“园区建设推进年”活动,加大园区基础设施、标准厂房建设和土地储备力度,支持旺高工业区物流大道、粤桂示范区合作大道、贺州华润循环新城(一期)基础设施等项目建设,力争纯一陶瓷、双文粉体等一批工业项目竣工投产。

　　持续抓好“长寿·养生”经济发展。主动对接粤港澳大湾区巨大的市场需求,发挥“世界长寿市”“中国十大养生城市”的金字招牌效应,加大养生旅游项目投入和基础设施建设,挖掘长寿文化资源和品牌,不断延伸长寿养生经济产业链条,着力抓好与南方传媒集团、港中旅等知名企业的合作,推动梦境黄姚文化旅游创意城、姑婆山足球产业园等项目的落地。加快国家全域旅游示范区建设,积极推进“四个特色小镇”项目和乡村旅游示范点建设,巩固提升昭平创建广西特色旅游名县工作成果,力争姑婆山旅游区成功创建国家“5A”级旅游景区、黄姚古镇通过国家“5A”级旅游景观质量专家评审,推进全国森林旅游示范市建设。加快国家“医养结合”试点市建设,推进姑婆山、东潭岭、园博园等医养结合项目,全力推进贺州市长寿养生产业园建设；抓好养老服务业改革试验区建设,加快医养结合综合服务体系示范项目的申报工作,扎实推进全区中医壮瑶医药养生保健示范基地建设；加快设立长寿办,筹建国际长寿博物馆,举办好贺州长寿高峰论坛,打响“生态贺州·长寿胜地”品牌。

　　加快发展现代服务业。深入推进我市国家服务业综合改革试点工作,加快研究出台促进服务业发展的政策措施。抓好重大服务业项目建设,力争2018年现代服务业重大项目新开工10项、续建22项、竣工投产1项,完成投资26亿元。深入实施“服务业综合改革专项行动”,建立服务业运行监管平台、中国石材碳酸钙大数据中心,加快建设平桂石材碳酸钙产品展销中心、贺州(深圳)智慧产业园·长寿谷等项目。以服务业集聚区建设为主要抓手,重点推进黄姚古镇旅游文化产业区、中国东盟石材碳酸钙交易中心集聚区等项目建设；加快培育国家服务业综合改革试点产业园。积极培育大物流,重点推进贺州市农产品配送中心和贺州农产品商贸园等项目,着力抓好马江临港物流园区、华砻物流配送中心等特色物流园区和配送体系建设。推动电子商务加快发展,深入推进富川、昭平电子商务进农村综合示范项目,不断完善农村电子商务服务体系。

　　培育壮大现代特色农业。实施农产品区域品牌战略,抓好粤港澳大湾区“菜篮子”“果篮子”和“肉篮子”基地建设,积极培育八步三华李、平桂水生蔬菜、钟山贡柑、富川脐橙、昭平茶等区域品牌和“天贺正丰”等企业品牌。大力推进现代特色农业示范区增点扩面提质升级,充分发挥和扩大八步区满天下李子产业示范区、平桂猪福天下生态养殖(核心)示范区、钟山幸福冲贡柑产业示范区、富川神仙湖果蔬产业示范区、昭平南山茶海示范区的辐射带动效应,加快建设一批农业产业园、科技园、创业园。不断延伸生态农产品产业链,培育和引进农产品龙头企业,加快农产品加工聚集区建设,加强与广西农科院、华南农业大学等院校的交流合作,促进科技成果转化,着力提升农产品精深加工和农产品附加值。利用“农业+”模式,推进农业与旅游、教育、科技、加工业、服务业等深度融合发展,促进农村一二三产业融合发展。发展土地流转、土地托管、土地入股等多形式适度规模经营,采用“公司(合作社)+基地+农户”模式,构建“产、供、加(工)、销”一体化产业发展格局,推动农业规模化经营、标准化生产、企业化管理。稳定粮食种植面积197万亩、产量71万吨,确保粮食安全。强化农产品质量安全监管,严格执行标准化生产和质量安全认证,健全农产品质量检测和追溯体系,提高动物疫情防控能力,积极开展“三品一标”建设与富硒农产品开发,不断提高全市农业品牌效应和价值,积极构建现代化农业产业体系。

　　(三)以更大魄力推进重大项目建设。今天的投资就是明天的动能,今天的项目就是明天的结构。要紧紧扭住项目建设不放松,强化土地、资金和服务保障,充分发挥项目投资对经济增长的支撑作用。

　　着力扩大有效投资。切实发挥项目建设指挥部和“10+2”议事协调机构的作用,落实厅级领导联系服务重大项目和“一把手”抓项目抓投资工作责任制。大力推进2018年市级层面统筹推进重大项目490项,力争完成投资300亿元以上,其中,新开工项目104项,续建项目178项,竣工投产项目58项。同时,抓好2017年新签尚未开工的97个项目落地,完成2018年招商引资到位资金同比增长10%以上。加大支柱产业和园区、景区的重大基础设施项目投资,重点抓好碳酸钙、装配式建筑产业等领域的投资,增强发展后劲。充分利用好国家重大建设项目库,精心谋划一批符合国家、自治区产业导向和投资政策的大项目、好项目,加快项目前期工作,争取更多项目纳入国家和自治区计划盘子。

　　着力推进基础设施建设。全力加快交通大通道建设。铁路方面,争取早日开工建设贺梧城际铁路；加快柳贺韶铁路、洛湛铁路(永州至玉林段)扩能改造、柳贺城际铁路、贺州动车组存车场等项目前期工作；公路方面,加大贺巴高速贺州段、永贺高速贺州支线、贺连高速贺州段、信梧高速贺州段等项目推进力度；力争望高至富川一级公路建成通车；开工建设南环路、西南环路,尽快形成城区环线；推进黄田至姑婆山等景区道路改造升级。机场方面,加快推进贺州支线机场和通用机场项目,启动贺州通用航空产业园项目。水运方面,加快贺江扩能一期工程前期工作,贺州临港产业区一期基础设施项目全面开工,力争实现贺州集装箱专用港口零的突破。

　　着力强化项目要素保障。强化用地保障,严格按照中央、自治区的时间节点要求做好用地报批和供地保障工作,确保完成项目用地报批500公顷以上、全市供地率达60%；攻克征拆难题,把征地拆迁列入县(区)党政“一把手”责任考核内容,完成中心城区三大片区、各工业园区土地征收及房屋拆迁安置工作；全力整合盘活项目用地,把土地调整给有资金、能开工的项目。拓宽项目融资渠道,密切跟踪中央预算内投资、政府债券、国家专项建设基金的投向,争取中央、自治区安排更多项目资金；大力开展政金企项目融资需求工作,加大外贷资金力度,争取发行企业专项债券；稳妥采取政府和社会资本合作(PPP)、政府购买服务等模式,引入民间资本进入公共领域投资；扎实做好投资存量资金清理和调整使用工作,充分发挥政府投资的引导作用。不断提升服务项目的水平,落实政务服务“三集中”制度,巩固完善“一个平台管项目审批”制度,提高规划审批、土地预审、环境评估等程序效率。

　　(四)以更大格局推进向东开放。深入实施“向东开放”战略,加快产业、资本和人才融入步伐,不断提高开放质量和水平,着力构建全方位开放合作新格局。

　　加快融入“两带一区”步伐。深化与粤桂黔高铁经济带合作试验区广东园、贵州园以及高铁沿线城市的合作,积极把握承接先进制造业转移和开展大数据产业合作的机遇,加快贵广高铁经济带合作示范区建设。主动融入和服务粤港澳大湾区建设,不断深化跨区域分工协作,以绿色农产品、健康养生养老和产学研合作为突破口,积极发挥粤港澳大湾区行业协会作用,在产业发展、项目引进、制度设计、政策安排等方面,实现与粤港澳大湾区共融互通。加快推进西江经济带基础设施大会战项目建设,推动贺州临港产业区规划建设,打通向东开放的水运通道节点。实施人才兴贺战略,加快专家村建设,大力发展会展经济,争取更多全国性、跨区域性的行业、协会和专业论坛举办地落户贺州,提升贺州在全区开放发展大局中的影响力。

　　全力促进精准有效招商。完成新签约项目投资480亿元。抓好园区平台招商,签订合作项目53个,投资总额118亿元以上。抓好产业招商和资本招商,重点面向粤港澳大湾区等发达地区,围绕培育壮大碳酸钙、装配式建筑和生态健康“三个千亿元产业”,切实发挥产业园区平台的承载作用,吸引总部经济、上市公司、龙头企业和优质项目落户贺州,推进我市传统行业改造升级和战略性新兴产业的发展。优化招商引资环境,加快制定完善产业扶持、园区经济发展的优惠政策,下大力气整治营商环境,健全政企沟通协商机制,充分发挥行业组织和乡贤资源的优势,增强服务企业的意识和水平,构建“亲”“清”新型政商关系。

　　大力发展外向型经济。鼓励国有平台公司深化跨区域合作,支持国有投融资平台围绕贺州优势产业“走出去”,主动对接产业发展的急需人才、技术、品牌和市场渠道,充分发挥平台公司在服务“三个千亿元产业”发展中的主力军和引领作用。加快贺州海关、马江对外开放口岸申报建设,促进税源回流；加快外贸综合服务平台建设,促进跨界电商、服务外包等新业态和中小型外贸企业的发展。继续实施“自主品牌出口增长行动计划”,支持碳酸钙和装配式建筑等优势产业走出去,鼓励利升岗石等企业申报“广西出口名牌”。

　　(五)以更大努力推动城乡统筹发展。牢固树立和践行绿水青山就是金山银山的理念,争创“山水林田湖草”综合治理示范市,加强美丽贺州建设。

　　加快绿色宜居城市建设。突显贺州山水、园林、长寿的特质,以“多规合一”试点工作为引领,完善城市总体规划修编、中心城区控制性详细规划。持续加快城区建设步伐,统筹推动城东新区、高铁新城、园博园新城、江南新城建设,深入推进灵峰山以西旧住宅整治改造工程,扎实推进贺州文化旅游产业综合开发项目建设。加快完善城市基础设施,稳步推进园博园、爱莲湖等公园二期建设；持续加快建设“一江两岸”和“海绵城市”,加大地下综合管廊推进力度；深入实施城区交通畅通工程、城区主次干道提质改造工程,加快建设衔接重要节点、工业园区和旅游景点的路网项目；继续开展城区绿化和亮化活动,争创“国家园林城市”和“国家历史文化名城”。提升城市管理水平,深入开展市容“五乱”整治、违法建设专项治理五年行动；建成城市规划管理信息平台和城市管理数字化平台,推动城市智慧化管理。

　　实施乡村振兴战略。深化农村土地制度和集体产权制度改革,巩固完善承包地“三权”分置制度、农村基本经营制度,完成农村土地承包经营权确权登记颁证工作。加强乡村规划,建立健全乡村振兴体制机制,狠抓示范引领,做强中心城镇,持续抓好贺街、黄姚全国特色小镇建设,推进公会、莲山等广西百镇建设示范工程。持续巩固“清洁乡村”“生态乡村”建设成果,深入推进“宜居乡村”建设,重点实施产业富民、服务惠民、基础便民专项活动,实施农村改厨、改厕5.93万户,建成150个新型宜居农村社区,启动“幸福乡村”建设。大力完善乡村基础设施,推动　“路、水、电、网、气、园”等重点领域实现新突破,全面完成乡镇“五小”项目建设任务。加强乡村治理,持续推进城乡社区协商,争创全国农村社区建设示范单位。支持和鼓励农民就业创业,拓宽增收渠道。

　　做大做强县域经济。园区是产业发展的重要载体,也是带动县域经济发展的强力引擎。五大工业园区和黄姚、姑婆山、临港三大产业区要围绕资源、交通、区位、产业等现有优势和潜在优势,完善发展规划,明确发展定位,优化产业布局,加强基础设施建设,强化精准有效招商,营造宜居宜业宜商环境,推动园区和产业区建设取得新成效,促进县域经济取得新发展。各县(区)要坚持产业强县、特色兴县,紧紧抓住自治区实施县域经济三年行动计划的机遇,着力在推动园区支柱产业、生态产业、农产品加工、全域旅游、健康产业发展等方面下功夫,不断增强县域经济发展动力和活力。八步区要围绕工业、商贸物流、旅游等方面,推进粤桂县域经济产业合作示范区、贺州现代商贸产业园等园区建设,加快建设向东开放发展先行区和贸易物流集聚区。平桂区要围绕碳酸钙全产业链、装配式建筑产业链,依托旺高工业区和生态产业园,加快园区科技服务中心、碳酸钙产品展示长廊等项目建设,积极打造碳酸钙、装配式建筑千亿元产业。钟山县要依托钟山工业园区,大力发展碳酸钙下游产品、轻工业、机械制造以及商贸物流业,加快建成贺州轻工业基地。富川瑶族自治县要围绕循环经济产业,继续壮大果蔬等特色农业,发展休闲农业与乡村旅游产业,加快国际慢城建设,打造全国循环经济示范区基地。昭平县要围绕生态健康产业,加快建设以黄姚古镇旅游文化产业区为龙头的全域旅游发展和医养结合示范区,全力打造旅游、茶叶、饮用水“三个百亿元产业”。

　　不断优化生态环境。实施工业废气废浆废渣治理,支持华润电厂和华润水泥厂固废处理项目建设,着力抓好城市扬尘污染治理。全面落实“河长制”和“湖长制”,扎实推进市污水处理厂扩建工程、工业园区污水处理厂以及贺州市水环境治理与城市综合发展等项目建设；继续推进路花水库、龟石饮用水源保护等工程建设,加快大湾水库饮用水源工程、黄姚片区水资源配置工程前期工作。抓好土壤污染治理和修复规划编制工作,加强重金属污染治理项目监督；确保中央环保督察组反馈问题的整改落实；完成第二次全国污染源普查工作。推进水土流失综合治理,做好地质灾害防治；加大矿山地质环境保护力度,加快推进平桂区大理石矿区复绿和排废区建设,抓好矿山历史遗留环境问题整治。

　　(六)以更大勇气深化改革创新。必须增强改革的主动性、坚定性和创造性,巩固改革成果,持续深化重点领域和关键环节改革,强化创新驱动发展,不断增强经济社会发展活力。

　　深化供给侧结构性改革。持续推动“三去一降一补”各项政策落地实施。严禁产能过剩行业违规新上项目,加快淘汰落后产能。坚持房子是用来住的、不是用来炒的定位,加强房地产市场运行监测和供需双侧调控,落实上级有关房地产市场调控政策,加大力度解决房地产历史遗留问题,促进房地产市场平稳健康发展。探索建立多主体供给、多渠道保障和租购并举的住房制度,继续推进城镇棚户区改造,加大对新市民的公租房保障力度。进一步强化政府债务监管、评估和预警,防范债务风险,做好政府债务消化工作。打好降本增效“组合拳”,减轻企业综合成本,积极申报参与广西电力市场化交易,抓好华润示范区增量配电业务试点工作。

　　推进重点领域和关键环节改革。持续深化“放管服”改革,继续推进简政放权,抓好“一站式服务”,清理规范行政审批及中介服务事项,推进“证照分离”改革,全面推进“双随机、一公开”监管,完成市县权责清单“两单融合”工作,优化行政权力运行流程。持续深化投融资改革,聚焦服务实体经济和产业发展等领域,重点加强工业园区和支柱产业的投资,激发经济内生动力；推进政府性融资担保体系建设,加快设立贺州市投资引导基金,支持各平台公司与社会资本设立股权类投资基金,不断拓宽融资渠道；加大对逃废金融债务的打击力度,严厉打击非法集资等违法违规金融活动。持续深化园区改革,充分发挥园区薪酬体制改革作用和招商引资政策优势,理顺园区与市本级和县(区)的税收分成制度,简化市管园区项目审批流程,释放园区发展活力。持续深化国资国企改革,改革国有资本授权经营体制,促进国有资产保值增值,理直气壮做大做强做优国有企业,推动市属国有企业横向联合、抱团取暖；采取多种方式剥离重组非主业资产,稳妥发展混合所有制经济,加快培育企业上市；强化国有企业内部考核管理与成本控制；加强国有企业监管,制定出资人监管权力和责任清单,授予企业完善的经营自主权；大力支持民营企业发展,营造企业家健康成长环境,弘扬优秀企业家精神,形成国有企业顶天立地、民营企业铺天盖地的格局。持续深化财税体制改革,继续推进预决算公开,增强政府理财工作的透明度；继续深化国库管理改革；全面实施绩效管理。探索成立村级土地资产管理公司,把村级土地纳入商业开发,以入股分红的模式破解征地拆迁难题,促进村民向市民转化,共享新型城镇化发展成果。持续深化人才体制机制改革,力争在体制外人才引进和柔性引进的政策体系上取得突破,围绕“三个千亿元产业”加大面向粤港澳大湾区引才的力度；建立完善人才评价和激励机制,激发人才干事创业活力。

　　全面实施创新驱动发展战略。继续加大研发投入,推动重点实验室基地建设,加快建设国家碳酸钙产品质量监督检验中心、广西碳酸钙资源综合利用重点实验室培育基地,以及微波技术应用、果蔬保鲜与深加工研究等自治区级实验室。以产业发展、技术入股和实体项目为纽带,加深与知名高校、科研院所合作,积极共建碳酸钙、非金属轻质材料、健康长寿等产业研究院。大力支持贺州高新技术产业开发区创建国家级高新技术园区,加快推进贺州华润循环经济产业示范区创建国家生态工业示范园区工作,积极推进国家农业科技园区建设。实现10项以上科技成果产业转化,力争申报自治区级创新驱动重大项目。鼓励企业或科研院所赋予科技人员更大自主权,提高科技人员转化收益分享比例。

　　(七)以更大担当增进民生福祉。深入贯彻以人民为中心的发展思想,不断满足人民日益增长的美好生活需要,使人民获得感、幸福感、安全感更加充实、更有保障、更可持续。

　　优先发展教育事业。继续实施第三期学前教育三年行动计划,开展幼儿园示范园创建、集团化办园试点工作,力争学前教育三年毛入园率达85%,普惠性幼儿园覆盖率达68%。全力攻坚义务教育均衡发展,确保八步区、昭平县通过国家达标验收。全面推进教育学区制管理改革,扩大优质资源总量。大力推进市中心城区8所新(迁)建中小学(幼儿园)、广西师大附中贺州外国语学校等项目建设,加快贺州市体育运动学校建设。扎实开展高中阶段教育普及攻坚工作,提高教学水平和教育质量。完善贺州职业学院基础设施,加快黄姚职业教育中心建设。扎实推进贺州学院向应用大学转型。

　　抓好就业创业和社会保障。促进高校毕业生、返乡农民工、建档立卡贫困户多渠道就业创业,实现全市城镇新增就业1.01万人,农村劳动力转移新增就业1.7万人次。加快平桂农民工创业园建设。深入实施城乡居民收入倍增计划,缩小城乡居民收入差距。健全工资支付监控和保障制度,加大力度治理拖欠农民工工资问题。加快全国首批社保标准化建设步伐,探索社保“一窗式”经办服务模式改革,稳步推进新农合和城乡居民医保整合。按时足额发放城乡特困人员供养金和城乡低保资金。完成保障性住房和棚户区改造住房2827套新建任务。

　　繁荣发展文化体育事业。持续抓好党的十九大精神学习宣传贯彻工作。推进贺州“四馆”和村级公共服务中心等设施建设,加快构建现代公共文化服务体系。强化文化遗产传承保护,加大潇贺古道沿线的古城镇、古村落等文化遗产保护和开发利用,扎实推进瑶族、壮族等多民族文化保护建设。加强文化市场监管,深入开展“扫黄打非”工作。大力完善体育基础设施,加快推进平桂、富川体育中心等项目。广泛开展全民健身活动,大力发展群众体育运动。

　　实施健康贺州战略。深化医药卫生体制改革,全面取消以药养医制度,加快建立现代医院管理制度。推进市级公立医院与县级以上医院建成紧密型医联体。加强基层医疗卫生服务体系建设,推进基层医疗卫生薪酬制度改革。加强计生服务管理工作,加大出生性别比综合治理力度,平抑出生人口性别失衡。加快老龄事业和产业发展。

　　打造社会治理新格局。持续强化社会主义核心价值观教育,加强诚信贺州建设。全面落实安全生产“党政同责”和“一岗双责”制度,加大重点领域隐患排查整治力度,坚决防范和遏制重特大安全事故。加强食品药品安全监管和综合治理,争创广西食品安全城市。争创全国民族团结进步示范市。深入推进“法治贺州”“平安贺州”建设,强化应急管理能力建设,抓好综治信访维稳,依法妥善处理各类突发性、群体性事件,着力构建立体化社会治安防控体系,不断提升群众安全感和满意度。

　　三、全面加强政府自身建设

　　深入贯彻新时代党的建设总要求,坚持党对政府工作的领导,全面加强政府自身建设,着力增强政府公信力和执行力,努力建设人民满意的服务型政府。

　　加快建设法治政府。坚持科学民主决策,着力构建决策科学、执行坚决、监督有力的权力运行机制。坚决服从市委的领导,自觉接受市人大的法律监督和政协的民主监督,坚持“三重一大”事项报告制度,把法律约束体现到政府工作全过程。虚心听取民主党派、工商联、人民团体和各界人士的意见建议,办好人大代表议案和政协委员提案。严格规范公正文明执法,加快制定城市管理执法体制改革实施方案,依法有效化解纠纷。加大全民普法力度,全力推进覆盖城乡居民的公共法律服务体系建设,全面提高政府工作人员法治思维和依法行政能力。

　　全面提升行政效能。加强政务服务基础设施建设,改善各级政务服务中心服务环境,完成市民服务中心、活动中心建设工作。加快推进“互联网+政务服务”,制定和公开实施“零跑腿”事项清单,优化公共资源交易流程,建立健全市、县、乡、村四级联动政务服务网络体系,初步实现市级与自治区平台公共资源信息互联互通和信息资源共享。大力推动决策、执行、管理、服务和结果“五公开”,及时回应群众关心关注的问题。大力开展“减证便民”专项行动,坚决取消各类无谓证明、循环证明,切实为群众生活办事提供便利。大力发扬“四专”精神,坚持专业办事,增强学习主动性实效性,不断提升执政本领,引进紧缺专业人才,提高人岗相适度,让专业的人办专业的事；坚持专心做事,专心致志、埋头苦干,做到“在岗一分钟,尽职六十秒”,努力争创一流业绩；坚持专注成事,始终保持专注发展的政治定力,以“钉钉子”精神,推动工作提速提质提效；坚持专攻难事,主动担重任、挑大梁,做到“事不避难,勇于担当”,决不能因为胜利而骄傲,决不能因为成就而懈怠,决不能因为困难而退缩,高质量完成既定目标任务。

　　持之以恒正风肃纪。牢固树立“四个意识”和“四个自信”,坚决维护习近平总书记的核心地位,坚定维护党中央权威和集中统一领导。严格执行党风廉政建设责任制,推进“两学一做”学习教育常态化制度化,深入开展“不忘初心、牢记使命”主题教育。建立健全廉政风险防控机制,贯彻落实中央八项规定精神和国务院“约法三章”,严格控制“三公”经费支出,充分发挥审计监督检查作用,积极履行党风廉政建设“一岗双责”。从严抓好中央巡视组“回头看”和自治区党委第六巡视组反馈问题的整改落实,坚持无禁区、全覆盖、零容忍,保持惩治腐败高压态势,严肃查处各类违法违纪案件,加大整治群众身边腐败问题力度。认真贯彻落实《贺州市干部干事创业容错纠错实施办法》,旗帜鲜明地保护忠诚干净、敢于担当、抓铁有痕的干部,树立让吃亏者吃香、有为者有位的用人导向,营造忠诚干净担当的政治生态。

　　各位代表,幸福都是奋斗出来的!成绩也是奋斗而来的!让我们紧密团结在以习近平同志为核心的党中央周围,高举习近平新时代中国特色社会主义思想伟大旗帜,在自治区党委、政府和市委的坚强领导下,全面贯彻落实党的十九大精神,不忘初心,牢记使命,群策群力,奋力前行,大力倡导“说干就干,干就干好”的工作作风,加把劲干、创造性地干,拙劲加巧劲地干,不负我们的梦想,不负伟大新时代,为加快实现贺州发展新定位、决胜全面建成小康社会而努力奋斗!
